# Network capacity with probit-based stochastic user equilibrium problem

**DOI:** 10.1371/journal.pone.0171158

**Published:** 2017-02-08

**Authors:** Lili Lu, Jian Wang, Pengjun Zheng, Wei Wang

**Affiliations:** 1 Faculty of Maritime and Transportation, Ningbo University, Ningbo, China; 2 National Traffic Management Engineering & Technology Research Centre Ningbo University Sub-center, Ningbo, China; 3 Jiangsu Province Collaborative Innovation Center for Modern Urban Traffic Technologies, Nanjing, China; 4 Lyles School of Civil Engineering, Purdue University, West Lafayette, Indiana, United States of America; Beihang University, CHINA

## Abstract

Among different stochastic user equilibrium (SUE) traffic assignment models, the Logit-based stochastic user equilibrium (SUE) is extensively investigated by researchers. It is constantly formulated as the low-level problem to describe the drivers’ route choice behavior in bi-level problems such as network design, toll optimization et al. The Probit-based SUE model receives far less attention compared with Logit-based model albeit the assignment result is more consistent with drivers’ behavior. It is well-known that due to the identical and irrelevant alternative (IIA) assumption, the Logit-based SUE model is incapable to deal with route overlapping problem and cannot account for perception variance with respect to trips. This paper aims to explore the network capacity with Probit-based traffic assignment model and investigate the differences of it is with Logit-based SUE traffic assignment models. The network capacity is formulated as a bi-level programming where the up-level program is to maximize the network capacity through optimizing input parameters (O-D multiplies and signal splits) while the low-level program is the Logit-based or Probit-based SUE problem formulated to model the drivers’ route choice. A heuristic algorithm based on sensitivity analysis of SUE problem is detailed presented to solve the proposed bi-level program. Three numerical example networks are used to discuss the differences of network capacity between Logit-based SUE constraint and Probit-based SUE constraint. This study finds that while the network capacity show different results between Probit-based SUE and Logit-based SUE constraints, the variation pattern of network capacity with respect to increased level of travelers’ information for general network under the two type of SUE problems is the same, and with certain level of travelers’ information, both of them can achieve the same maximum network capacity.

## 1. Introduction

Network capacity aims to describe the maximum demand that can be accommodated by the road network. It can be measured by the greatest common multiplier of existing origin-destination (O-D) demand that can be achieved without exceeding a prescribed degree of saturation on each links while taking users’ route choice into account [1]. Like the indicators such as travel time, queue length etc., network capacity is also a very important indicator to evaluate the network performance. For example, on infrastructure planning, an underlying guideline to design the road network and determine the number of lanes on particular link is that they should have enough capacity to accommodate the demand in future. One advantage of study of network capacity is, it helps to find the potential links that may block the traffic, and determine the optimal investment strategy to improve the total capacity of the network. Besides, for congested urban traffic network, it is also able to relieve traffic congestion level by determining optimum inputs such as the signal timings (circle time, splits etc.), link capacity increase etc.

Wong and Yang [2] first proposed the concept of reserve capacity of a general signal-controlled road network under time-stationary conditions with deterministic user equilibrium (DUE) problem. It is defined as the maximum common multiplier of existing O-D demands that the network can accommodate under certain constrains. They designed a bi-level programming model to describe the network reserve capacity problem and proposed a heuristic algorithm based on sensitivity analysis of DUE problem to find the optimal settings of signal splits to maximize the network capacity. This concept is redefined by Gao and Song [3]. It found that if the multipliers of different O-D demand could be varied independently, the network can achieve larger capacity under the same condition proposed by Wong and Yang[2]. Chiou [4] studied the network capacity with optimal signal setting problem, it is formulated as bi-level mathematical program and a projected gradient approach was proposed to solve this problem. To find the optimal number of lanes assigned to each flow direction in a road, Wang and Deng [5] studied the network capacity problem with reversible lanes. It is formulated as a bi-level programing problem where low-level problem is a DUE problem proposed to describe the drives’ route choice, and the upper-level problem is a mixed integer programing problem aiming to find the optimal signal settings (i.e., signal circle and signal splits) and number of lanes in each direction of a reversible road to maximize the network capacity.

The pre-mentioned literatures all studied the network capacity problem at the DUE condition. In order to overcome the unpractical assumptions associate with DUE (i.e., all drivers have perfect knowledge of traffic condition and choose the paths with minimum cost between corresponding O-D pair), numerous literatures extended the research of network capacity with Logit-based SUE problem where the travelers are assumed to make route choice to minimize their perceived travel cost (see e.g., [6–9]). They found that the network capacity at SUE state is comparatively larger than it is at DUE when provided certain level of traveler’s information. Besides, Wang *et al*. [7] also reveals that with the same total budget to improve the capacity of links in the network, the resulted optimal network capacity at SUE condition is larger than that at the DUE condition. The other studies in this branch could be found by Chootinan *et al*. [10] which takes the link capacity as a random variable and study the probability of a certain level of traffic demand that the road network can accommodate at SUE condition.

It should note that while some researchers notice the unpractical assumption of DUE problem and use the Logit-based SUE problem, the Logit-based SUE problem still can’t realistically capture the travelers’ route choice behavior. Due to the independent and irrelevant alternatives (IIA) assumption, the Logit-based SUE problem has an inherent defect that it lacks of sensitivity to network topology and assigns too much flow on the overlapped routes. Besides, the route choice probabilities given by Logit-based SUE problem only depend on absolute difference of route travel time and take no account of the relative difference which is more important [11]. Generally, compared with Logit-based SUE models, the traffic assignment result provided by the Probit-based model is believed to be more consistent with the real-world observation, which overcomes the IIA problems stated above. However, few researches so far have been done considering the network capacity with Probit-based SUE problem. As a matter of fact, to the authors’ knowledge, few researches incorporate the Probit-based SUE problem as the route choice model to study the bi-level network design problem. One possible reason is perhaps because the Probit-based SUE problem has no close-form formulation of route choice probabilities and is hard to solve for the optimal solution (usually Monte Carlo technique is applied), which increases the difficulty to design the solution algorithm for the bi-level programming problem. Notice that a traffic assignment model that better captures driver route choice behavior is critical for practical value (such as optimal settings of signal splits, link capacity expansion etc.) of network design problems, the Probit-based SUE problem deserves more attention.

This paper continues our previous research of network capacity [7] by assuming that the drivers all make their route choices based on Probit-based SUE principles. A bi-level programming is formulated to describe the network capacity with Probit-based SUE problem, and a heuristic solution algorithm based on sensitivity analysis of the Probit-based SUE problem are proposed to solve the bi-level programming problem. The relationship between network capacity and the quality of driver’s information is explored subsequently. Moreover, the maximum network capacity at both Probit-based and Logit-based SUE condition is explored and the differences between them in certain network topology are examined and demonstrated with a few small numerical examples.

The contributions of this study are twofold: first, we studied the road network capacity by incorporating Probit-based SUE model, the assignment result of which is more consistent with the real world. We also propose a SAB method to solve the bi-level network capacity with Probit-based SUE problem. It can efficiently find the optimal settings of network inputs (such as signal splits, link capacity expansion etc.) to maximize the network capacity. Second, we compare the variation pattern of network capacity when level of drivers’ information changes. The level of drivers’ information in previous study [1,7] is found to be an important parameter that significantly impacts the network capacity. This study demonstrates with theoretical analysis that when level of drivers’ information changes, the variation pattern of network capacity between Logit-based SUE and Probit-based SUE constraints are the same. This study also finds that perfect knowledge of traffic condition may not contribute to network capacity. The network capacity achieves the maximum value when travelers’ information is controlled at certain level. This is because non-perfect information motivates travelers to use the additional capacity on some links more effectively. The findings can assist transportation planners to design policies to relieve the traffic congestion.

The remainder of this article is structured as follows: in the next section, the network capacity with Probit-based SUE problem is discussed and formulated. In Section 3, a heuristic method based on sensitivity analysis for SUE problem is explicitly presented to solve the proposed bi-level network capacity problem. Section 4 presents three numerical examples to illustrate the general application of proposed methods for network capacity problem and comparison is conducted between network capacity with Probit-based and Logit-based SUE problem. The last section concludes the paper.

## 2. Reserve capacity with Probit-based SUE problem

At equilibrium state, the link flow is perturbed by demand multipliers vector **u** and a vector of perturbed parameters (such as link capacity, free flow travel time). Consider a signalized road network, the signal timings in the intersections impact the maximum traffic flow that can go through in one unit time (for example, one hour). Thereby, they can be seen as the perturbed parameters that can affect the equilibrium link flow. Let **λ** denote the vector of signal splits. Then the link flow could be formulated as a function of the O-D demand multipliers and signal splits. In order to ensure the delays and queues are acceptable at the equilibrium condition, the link flows that approach to the signalized intersection must satisfy capacity constraint given as follows:

$$v_{a}(u,\lambda )\leq \rho_{a}s_{a}(\lambda_{a})\;a\in A$$ (1)

where $\bar{A}$ is the set of links in the network; *ρ*_*a*_ is the preset maximum saturation rate on link *a*, *a* ∈ *A*; *λ*_*a*_ is the signal split of link *a*; s_*a*_(*λ*_*a*_) denotes the capacity of link *a*, it is a function of signal split *λ*_*a*_; In addition to the link flow constraint, the green time at signal-controlled intersections and O-D demands should satisfy some linear conditions, given as follows:

$$\lambda_{\text{min}}\leq \lambda_{a}\leq \lambda_{\text{max}}\;a\in \bar{A}$$ (2)

$$\mu_{rs}\geq \mu_{0}\;\forall r,s$$ (3)

where $\bar{A}$ is the set of signalized links in the network. *λ*_min_ is the minimum green split, *λ*_max_ is the maximum green split. *r* is an origin node and *s* is a destination node. *μ*_0_ is the minimum O-D demand multiplier. For simplicity, the signal lost time is not considered in the paper. Thus, green split would satisfy the following relationship:

$$\sum_{n=1}^{N_{j}}\lambda_{nj}=1\;j\in J$$ (4)

where *J* is the set of all signalized intersections in the network; *j* is an signalized intersection on the road network, *j* ∈ *J*; *N*_*j*_ denotes the preset number of phases on signalized intersection *j*.

In this study, we use the definition of reserve capacity given by Gao and Song [3], that is, the demand multipliers between each O-D pair could be different. This concept relaxes requirement of common multiplier in Wong and Yang [2] by allowing the maximum throughput to be scaled by individual O–D pairs [12]. Under this definition, the mathematical programming for network capacity with SUE problem is formulated as follows:

$$\text{max }z=\sum_{\forall r,s}u_{rs}q_{rs}^{0}$$ (5a)

$$s.t\left\{ \begin{array}{l} v_{a}(u,\lambda )\leq p_{a}s_{a}(\lambda_{a})\;a\in \bar{A}\\ \mu_{rs}\geq \mu_{0}\;\forall r,s\\ \sum_{n=1}^{N_{j}}\lambda_{nj}=1\;j\in J\\ \lambda_{\text{min}}\leq \lambda_{a}\leq \lambda_{\text{max}}\;a\in \bar{A} \end{array}\right.$$ (5b)

where *u*_*rs*_ denotes the O-D multiplier of O-D pair *r-s*, $q_{rs}^{0}$ is the initial O-D demand between O-D pair *r-s*; $u_{rs}q_{rs}^{0}$ is multiplied O-D demand. *v*_*a*_(**u,λ**) is obtained by solving the following equivalent Probit-based (also for Logit-based SUE problem) SUE problem (Sheffi, 1985)

$$\text{min }Z=\sum_{a\in A}v_{a}t_{a}(v_{a},\lambda_{a})-\sum_{a}\int_{0}^{v_{a}}t_{a}(x,\lambda_{a})\text{d}x-\sum_{r,s}u_{rs}q_{rs}^{0}S_{rs}(c^{rs}(v))$$ (5c)

where *v*_*a*_ denotes the flow on link *a*; *t*_*a*_ is the travel cost on link *a*; *λa* is the signal splits of link *a*; *S*_*rs*_(**c**^*rs*^(**v**)) denotes the expected perceived travel between O-D pair *r* − *s*. It is a function of travel time of all routes between O-D pair *r* − *s*.

## 3. Solution algorithm for network capacity problem

### 3.1 Sensitivity analysis based (SAB) algorithm for bi-level network capacity problem

Due to the intrinsically non-convexity, the bi-level programing problem (5) is very difficult to solve for a globally optimal solution. Besides, the functional term *v*_*a*_(**u, λ**) in upper level problem (5b) is implicit, which can only be obtained by solving the Probit-based SUE problem. This increases the difficulty to direct solve the upper-level problem. In literature, the bi-level network capacity problems are generally solved with genetic algorithm (GA) (see e.g., [1, 5, 6, 13, 14]). However, GA is a random search technique that optimizing the solutions based on nature selection, it converges very slowly when high accuracy is required, thus is computationally expensive. Compared with GA, the SAB algorithm is much more preferable. It searches the optimal solution along the direction that the object function is minimized. The SAB method is previously used extensively for network capacity with DUE problem [2, 3]. The efficiency and applicability of this method in solving various bi-level optimization problems in transportation domain are also explored in many literatures [5, 9, 15–18]. In this study, we will use the SAB algorithm to solve the proposed network capacity problem (5).

The main idea of SAB method is to use a linear function to approximate the nonlinear and implicit function of equilibrium link flow *v*_*a*_(**u, λ**) in the upper-level program (5b). To achieve this, the derivatives of equilibrium link flows with respect to perturbed parameters (i.e. O-D demand multipliers, signal splits) should be obtained in advance. Assume the derivations have been calculated at current feasible point(v*, u*, λ*), then, according to the first-order Taylor approximation, the implicit functional form *v*_*a*_(**u, λ**) can be estimated as

$$v_{a}(u,\lambda )\approx v_{a}(u^{*},\lambda^{*})+\sum_{a\in \bar{A}}\frac{\partial v_{a}(u^{*},\lambda^{*})}{\partial \lambda_{a}}(\lambda_{a}-\lambda_{a}^{*})+\sum_{\forall r,s}\frac{\partial v_{a}(u^{*},\lambda^{*})}{\partial u_{rs}}(u_{rs}-u_{rs}^{*})$$ (6)

Substituting Eq (6) into the upper-level problem (5b), the upper-level problem will become an ordinary linear programming problem with the variable signal splits and O-D demand multipliers. This ordinary problem can be solved by the simplex method, thus one can get a new improved point (v*^1^, u*^1^, λ*^1^) from which a new linear programming problem is again generated and can be again solved by the same method. Repeat the steps, the algorithm converges to an optimal solution. Denote **v** as the vector of all link flows. The steps for implementing SAB method to solve the bi-level network capacity problem (5) are summarized as follows:

*Step 1* Determine an initial set of the values (u*, λ*). Set *n* = 0.
*Step 2* Using method of successive averages (MSA) [11] to solve the lower-level SUE problem for given **u**^*n*^, and **λ**^*n*^ and hence get **v**^*n*^, ∀ *a* ∈ *A*.
*Step 3* Calculate the derivatives ∂**v**/∂**u**, and ∂**v**/∂**λ** with the sensitivity analysis method for Probit-base SUE problem.
*Step 4* Formulate local linear approximations of the upper-level link flow term *v*_*a*_(**u**, **λ**) with the derivative information, and use simplex method to solve the resulted linear programming to obtain the new O-D demands multipliers vector **u**^*n*+1^ and signal splits vector **λ**^*n*+1^.
*Step5* If max|(**u**^*n*+1^ − **u**^*n*^)/**u**^*n*^| ≤ *ε*_1_, and max|(**λ**^*n*+1^ –**λ**^*n*^)/**λ**^*n*^| ≤ *ε*_2_, then stop, where *ε*_1_, *ε*_2_ are predetermined tolerance. Otherwise let *n = n* + 1 and return to *Step 1*.

The main difficulty to solve problem (5) with the above steps is to obtain the derivatives of equilibrium link flow with respect to signal splits $\lambda_{a},\forall a\in \bar{A}$ and O-D multiplier *μ*_*rs*_,∀*r*,*s* This could be done by operating the sensitivity analysis of Probit-based SUE problem (i.e., problem (5c)). Sensitivity analysis is to measure how much the target objective would be alternated by one unit change of the explanatory variable. Sensitivity analysis for traffic assignment model could be dated back to the work done by Tobin and Friesz [19]. They developed the analytical formulation to obtain the gradient of equilibrium link flow with respect to perturbed parameters at DUE state. By using the same method, the formulation for sensitivity analysis of DUE problem with elastic demand problem is studied in [20]. Wang *et al*. [21] derived the formulation for second-order sensitivity analysis of DUE problem. Du *et al*. [22] discussed the analytical approach for sensitivity analysis of equilibrium trip distribution–assignment model with variable destination costs. Ying and Miyagi [23] formulated a computationally efficient link-based algorithm for sensitivity analysis of Logit-based SUE by adopting Dial’s algorithm [24]. This method is incorporated in the SAB algorithm to solve the bi-level network capacity with Logit-based SUE problem proposed in [7]. However, it is not applicable here since the low-level problem of the proposed network capacity problem is Probit-based SUE instead of Logit-based SUE. Clark and Watling [25, 26] develop another sensitivity analysis method for SUE problem by adopting first-order sensitivity approximation of a general nonlinear program proposed by Fiacco [27]. This method is capable to obtain derivative of equilibrium link flows with respect to perturbation parameters at both Logit-based and Probit-based SUE condition. Hence, it is suffice to calculate derivatives of equilibrium link flows with respect to signal splits $\lambda_{a},\forall a\in \bar{A}$, and O-D multiplier *μ*_*rs*_,∀*r*,*s* in Eq (6). For completeness, in the following, we will briefly present the procedures for sensitivity analysis with Probit-based SUE problem.

### 3.2 Sensitivity analysis for Probit-based SUE

Given a nonlinear programming formulation *p*_3_(*ε*), where *ε* is a disturbed parameter which provide small changes in formulation of the objective function or the constraints,

$${\text{min}}_{\text{x}}\;z(\text{x},\varepsilon )$$

s.t

$$\begin{array}{l} g_{i}(\text{x},\varepsilon )\geq 0\;(i=1,\cdots ,m)\\ h_{j}(\text{x},\varepsilon )=0\;(j=1,\cdots ,n) \end{array}$$

Let the *u*_*i*_ be the Lagrange multiplier for inequality constraint *g*_*i*_(**x**, *ε*), and *w*_*j*_ be the Lagrange multiplier for inequality constraint *g*_*i*_(**x**, *ε*). Formulate this nonlinear program as the equivalent Lagrangian expression

$$L(\text{x},u,w,\varepsilon )=z(\text{x},\varepsilon )-\sum_{i}\mu_{i}g_{i}(\text{x},\varepsilon )+\sum_{j}w_{j}h_{j}(\text{x},\varepsilon )$$

If the nonlinear programming satisfies the four conditions explicitly presented by Fiacco (1983) for implementing the first-order sensitivity approximation (for details, see Clark and Watling (2001, 2002)), the sensitivity of the solutions and Lagrangian multipliers with respect to disturbed parameter *ε* then can be calculated by:

$$\left[\begin{array}{l} \nabla_{\varepsilon }\text{x}\\ \nabla_{\varepsilon }\text{u}\\ \nabla_{\varepsilon }\text{w} \end{array}\right]=-\text{M}{\text{(0)}}^{\text{-1}}\text{N}(0)$$ (7)

where ∇_*ε*_**x** is the derivatives of solutions with respect to parameter *ε* (dimension *n*); ∇_*ε*_**μ** is derivate of the m-vector of Lagrangian nonnegative multipliers with respect to *ε*; ∇_*ε*_**w** is the derivative of n-vector of Lagrangian equality multipliers with respect to parameter *ε*. The matrices M and N (as functions of *ε*) are given by:

$$M(\varepsilon )=\left[\begin{array}{ccccccc} \nabla^{2}L & -\nabla g_{1}^{T} & \cdots & -\nabla g_{m}^{T} & \nabla h_{1}^{T} & \cdots & \nabla h_{p}^{T}\\ \mu_{1}\nabla g_{1} & g_{1} & & 0 & & & \\ \vdots & & \ddots & & & 0 & \\ \mu_{m}\nabla g_{m} & 0 & & g_{m} & & & \\ \nabla h_{1} & & 0 & & & & \\ \vdots & & & 0 & & 0 & \end{array}\right]$$

$$N(\varepsilon )={\left[\begin{array}{ccccccc} -\nabla_{x\varepsilon }^{2}L^{T} & -\mu_{1}\nabla_{\varepsilon }g_{1}^{T} & \cdots & -\mu_{m}\nabla_{\varepsilon }g_{m}^{T} & -\nabla_{\varepsilon }h_{1}^{T} & \cdots & -\nabla_{\varepsilon }h_{p}^{T} \end{array}\right]}^{T}$$

Clark and Walting [25, 26] proved that the Probit-based SUE model (5c) satisfies the four conditions for implementing the first-order sensitivity approximation. Hence, Eq (7) can be adopted to calculate the derivative of equilibrium link flow with respect to perturbation parameters at Probit-based SUE state. Recall that problem (5c) is an unconstraint nonlinear program. Thereby, most of the terms in matrices of M and N vanish. The only term remained in matrix **M**(**ε**) and **N**(**ε**) are ∇^2^*L* and ∇^2^_v**ε**_*L*, respectively. The explicit expression for ∇^2^*L* is given as:

$$\nabla^{2}L=\nabla_{v}^{2}Z=\sum_{r,s}u_{rs}q_{rs}^{0}\left[(\nabla_{v}t\Delta^{rs})(-\nabla_{c}P^{rs}){\left(\nabla_{v}t\Delta^{rs}\right)}^{T}\right]+\nabla_{v}t\text{+}\nabla_{v}^{2}t\cdot \text{R}$$ (8)

where ∇_c_**P**^*rs*^ is Jacobian of the route choice probability vector for O-D pair *r*-*s*; **Δ**^*rs*^ is the link-route incidence matrix for O-D pair *r-s*; **t** is a vector of all link travel time; ∇_v_**t** denotes the Jacobian matrix of link travel time with respect to link flow; R is a diagonal matrix, the *a*th (∀*α*) element in the main diagonal is $\sum_{r,s}\sum_{k}q_{rs}P_{k}^{rs}\delta_{a,k}^{rs}+v_{a}$. In Eq (8), the method to calculate the Jacobian of the route choice probability vector for O-D pair *r*-*s* (i.e., ∇_c_**P**^*rs*^) could be found in [25, 26].

If the perturbed parameters are the O-D multipliers, then

$$\nabla_{v\mu }^{2}L=\nabla_{v\mu }^{2}Z=-\sum_{r,s}\left[q_{rs}^{0}P^{rs}\Delta^{rsT}\right]\nabla_{v}t$$ (9)

If the perturbed parameters are the signal splits, then

$$\begin{array}{l} \nabla_{v\lambda }^{2}L=\nabla_{v\lambda }^{2}Z\\ \;=-\sum_{r,s}u_{rs}q_{rs}^{0}\left[\left(\nabla_{v}t\Delta^{rs})(-\nabla_{c}P^{rs})\nabla_{\lambda }c^{rs}(\lambda \right)\right]\nabla_{v}t \end{array}$$ (10)

The derivative ∂*v*_*α*_/∂*u*_*rs*_,∀*α*, *r*, *s* in Eq (6) can then be calculated with Eqs (8), (9) and (7) (note **M**(ε) = ∇^2^*L* and $N(\varepsilon )=\nabla_{v\lambda }^{2}L$ in this context) and the derivatives $\partial v_{a}/\partial \lambda_{a},\forall a\in \bar{A}$ can be obtained with Eqs (8), (10) and (7) (note $N(\varepsilon )=\nabla_{v\lambda }^{2}L$ in this case).

## 4. Numerical examples

### 4.1 Numerical example 1

**Fig 1:**
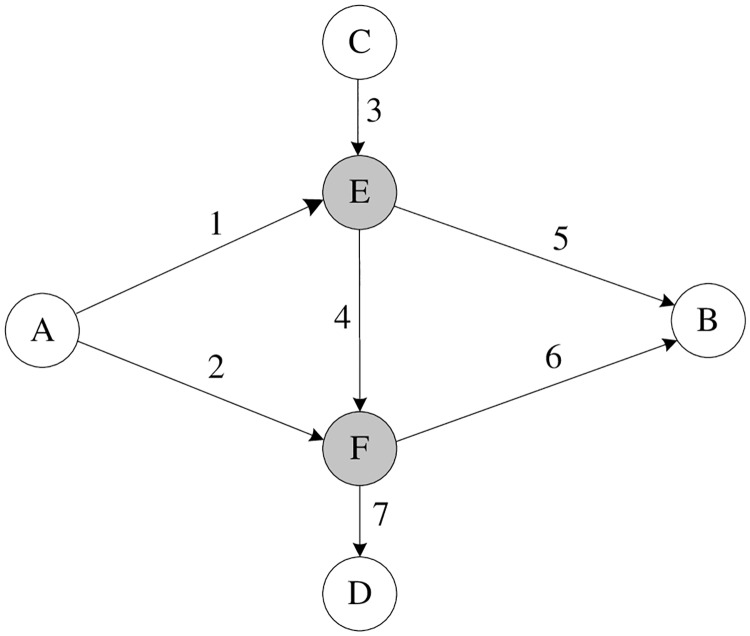
The example road network.

For comparison, the same numerical example network shown in Fig 1 is adopted in this study. It is originally used by Wang *et al*.[7] to demonstrate the network capacity with Logit-based SUE constraint. This numerical example network contains two O-D pairs, seven links and six nodes, where nodes *E* and *F* are signal-controlled intersections. There are three paths for the O-D pair A-B, i.e., route 1: *AEB*; route 2: *AFB* and route 3: *AEFB*, while there is only one path, *CEFD* for O-D pair C-D. The current O-D demand form nodes A to B is 18 veh/min, and from nodes C to D is 6 veh/min. The input data taken from Gao and Song [3] is summarized in Table 1. Suppose intersections *E* and *F* are controlled by two independent splits, *λ*_1_ and *λ*_2_ Signal control variables for link 1, 2, 3 and 4 are represented by *λ*_1_, *λ*_2_, *λ*_3_, *λ*_4_. The lower and upper bounds of signal splits are 0.05 and 0.95, respectively. The maximum degree of saturation for all signal-controlled links is set the same as *P* = 0.9. The expected link travel time is a random variable that is assumed to be normally distributed with mean equals to the link travel time and with variance that is proportional to the measured link travel time. Namely,

$$T_{a}∼N(t_{a},\alpha t_{a})$$ (11)

where *N*(·) represents the normal distribution. *α* is the variance of the perceived travel time (for one unit) over a road segment. Under this assumption, the covariance of route travel time is then subjected to the following multivariate normal distribution:

$$C_{rs}∼MVN(t\Delta^{rs},\alpha \Delta^{rs}t\Delta^{rsT})$$

**Table 1:** Input data to the example network.

Link number *α*	1	2	3	4	5	6	7
Free-flow time $t_{a}^{0}$	2.0	1.0	2.0	3.0	1.0	2.0	1.0
Saturation flow *s*_*α*_	24	30	30	35	24	30	30
Link travel cost:	$t_{a}(v_{a},\lambda_{a})=t_{a}^{0}{\left[1.0+0.5(v_{a}/\lambda_{a}s_{a}\right)}^{2}]$						

The Probit-based SUE problem is generally solved with MSA method. However, unlike the Logit-based model, the Probit-based SUE problem does not have an explicit function to represent the route choice probabilities. Thereby, it is generally solved with Monte-Carlo simulation technique, where slight difference may exist for the solutions obtained by running the simulation technique several times. This may impose some perturbations to the SAB method, making it hard to converge. To address this problem, we fixed the standard normal sampling data in Monte-Carlo simulation process and use the same sampling data in different iterations. With this operation, the SAB method is able to give the same solutions with the same inputs. The numerical result obtained at each iteration of SAB method at *α* = 1 is summarized in Table 2. It shows that the SAB method converges after four iterations, very efficient to calculate the optimal solution for the network capacity problem (5).

**Table 2:** Numerical results for network capacity problem with SAB method at *α* = 1.

Iterations	*λ*_1_	*λ*_2_	*μ*_*AB*_	*μ*_*CD*_
1	0.500	0.500	1.000	1
2	0.778	0.801	2.043	1
3	0.778	0.801	2.024	1
4	0.778	0.804	2.024	1

**Fig 2:**
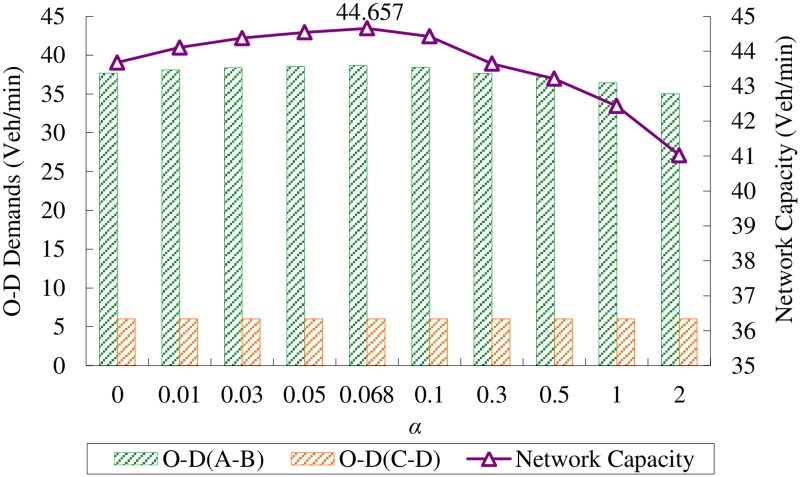
O-D demands and network capacity at Probit-based SUE conditions with different *α*.

It deserves mentioning that the parameter *α* in Eq (11) represents the level of travelers’ information. When *α* increases, the variance of the perceived link travel time will increase, implying that the quality of travelers’ information is reduced. Table 3 demonstrates the optimal signal splits, O-D multiplies as well as equilibrium link flows when network capacity is achieved with different *α*. It shows that when *α = 0*, at which condition the SUE problem turns into be DUE problem, the corresponding optimal O-D multiplies are *u*_*AB*_ = 2.093;*u*_*CD*_ = 1, the same as it is calculated by Gao and Song [3]. When *α* increases, the O-D multiplier *u*_*AB*_ perturbs slightly while the O-D multiplier *u*_*CD*_ is always fixed, contribute little to the varied network capacity. Fig 2 describes the maximum network capacity and optimal O-D demands between O-D pair A-B and C-D with different *α*. We can see that the network capacity first increases monotonic with respect to increased *α* until it reaches the maximum value at *α* = 0.068, then it decreases monotonously as *α* continue to increases. In others words, the network capacity increases when the level of travelers’ information increases within certain range, and then decreases if more information is provided. It worth mentioning that network capacity is not maximum at the DUE state where the drivers have perfect knowledge of traffic condition. This is because better information allows a large portion of demand to use the fast route, thus saturates the weakest link of that route, making it impossible to accommodate more traffic.

**Table 3:** Numerical results for problem (5) with different *α*.

α	0	0.01	0.03	0.05	0.068	0.1	0.3	0.5	1	2
*λ*_1_	0.778	0.778	0.778	0.778	0.778	0.778	0.778	0.778	0.778	0.778
*λ*_2_	0.810	0.81	0.81	0.81	0.81	0.81	0.81	0.809	0.804	0.781
*λ*_3_	0.222	0.222	0.222	0.222	0.222	0.222	0.222	0.222	0.222	0.222
*λ*_4_	0.190	0.190	0.190	0.190	0.190	0.190	0.190	0.191	0.196	0.219
*u*_*AB*_	2.093	2.117	2.132	2.141	2.148	2.135	2.091	2.067	2.024	1.946
*u*_*CD*_	1.000	1.000	1.000	1.000	1.000	1.000	1.000	1.000	1.000	1.000
*v*_1_	15.820	16.251	16.518	16.685	16.799	16.8	16.8	16.8	16.8	16.8
*v*_2_	21.857	21.857	21.857	21.857	21.855	21.628	20.844	20.412	19.633	18.224
*v*_3_	6.000	6.000	6.000	6.000	6.000	6.000	6.000	6.000	6.000	6.000
*v*_4_	6.000	6.000	6.000	6.000	6.000	6.000	6.0002	6.001	6.175	6.900
*v*_5_	15.820	16.251	16.518	16.685	16.799	16.8	16.8	16.791	16.625	15.900
*v*_6_	21.857	21.857	21.857	21.857	21.855	21.628	20.844	20.421	19.808	19.124
*v*_7_	6.000	6.000	6.000	6.000	6.000	6.000	6.000	6.000	6.000	6.000

**Fig 3:**
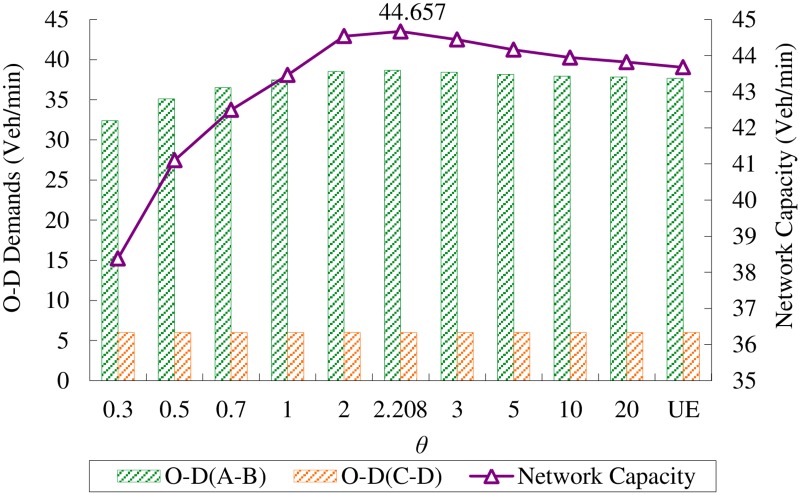
O-D demands and network capacity at Logit-based SUE conditions with different *θ*.

In order to make a comparision, the road network capacity at Logit-based SUE condition is presented in Fig 3. These results are cited from Wang *et al*. [7]. Denote *θ* as the dispersion parameter in the Logit-based SUE. Fig 3 shows that the network capacity firstly increases monotonously with respect to *θ* and gets the best performance at *θ* = 2.208, then it decreases slowly when *θ* continue to increase. Since the parameter *θ* in Logit-based SUE is a monotonic increasing function of the level of travelers’ information, the perturbed pattern of network capacity with respect to level of traveler’s information at Logit-based SUE condition is the same as it is at Probit-based SUE condition, i.e., the network capacity increases when provide the travelers with better quality of information, and after it researches the maximum, better quality of information will decrease the network capacity. Figs 2 and 3 also reveals that, for both Logit-based SUE and Probit-based SUE, network capacity has the same maximum value (i.e., 44.657) veh/min when the traveler’s information is controlled at certain level.

It should note that while the parameter *θ* and *α* represent the level of driver’s information in Logit-based and Probit-based SUE, respectively, there is no criteria to quantitatively decide the value of *θ* and *α* explicitly under the given level of driver’s information. Thereby, we cannot compare difference of the network capacity between the Probit-based SUE and Logit-based SUE under the same level of driver information. Nevertheless, as the route choice provided by Probit-based SUE is more consistent with the real world observations than Logit-based SUE, the network capacity obtained by this study is more practical.

### 4.2 Numerical example 2

**Fig 4:**
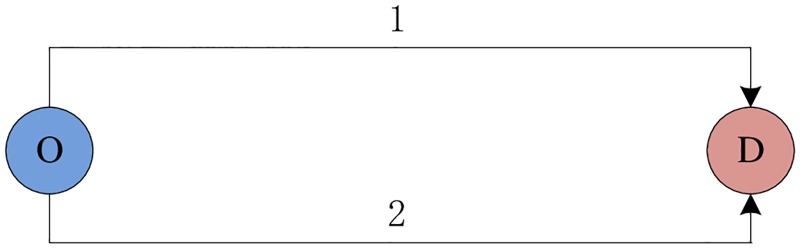
Numerical network for example 2.

Numerical example 1 demonstrates that the variation pattern of network capacity with respect to level of travelers’ information at Probit-based SUE state is the same as it is at Logit-based SUE state, and if provided certain quality of travelers’ information, they can reach the same maximum value. An underlying question in this context is whether this phenomenon is common for a general network or is just restricted to the numerical example 1. Ge *et al*. [1] pointed out that the link capacities as well as free flow travel time significantly impact the variation pattern of network capacity when level of travelers’ information changes. In this example, we assume the link capacities and free flow travel time are variable, and demonstrate how the network capacity changes with respect to link capacity and free flow travel time at Probit-based SUE condition. Due to the difficulty to directly formulate the explicit expressions of network capacity with respect to those perturbed parameters, we just demonstrate with a small network depicted in Fig 4 given by Ge *et al*. [1].

Fig 4 is a very simple network with only one O-D pair and two links. The link travel time is used as the BPR function as following:

$$t_{a}=t_{a}^{0}\left[1+\tau {\left(\frac{v_{a}}{s_{a}}\right)}^{\beta }\right]$$ (12)

Like numerical example 1, the covariance of expected perceived link travel time is assumed to be proportional to average link travel time, and the scale factor is *α*, the distributional function for route *R*_1_ and route *R*_2_ are:

$$R_{1}∼N\left(t_{1},\alpha t_{1}\right)$$

$$R_{2}∼N\left(t_{2},\alpha t_{2}\right)$$

The route choices probabilities for route 1 and route 2 are formulated as:

$$P_{1}=\text{Pr}(R_{1}<R_{2})=\text{Pr}(R_{1}-R_{2}<0)$$

$$P_{2}=\text{Pr}(R_{2}<R_{1})=\text{Pr}(R_{2}-R_{1}<0)$$

Since there is no overlap between route 1 and route 2, therefore, the covariance of *R*_1_, *R*_2_ is zero (i.e., cov(*R*_1_, *R*_2_) = 0). Then we have

$$R_{1}-R_{2}∼N(t_{1}-t_{2},\alpha (t_{1}+t_{2}))$$ (13a)

$$R_{2}-R_{1}∼N(t_{2}-t_{1},\alpha (t_{2}+t_{1}))$$ (13b)

According to Eq (13a) and (13b), we have

$$P_{1}=\Phi \left(\frac{t_{2}-t_{1}}{\sqrt{\alpha (t_{1}+t_{2})}}\right)$$ (14a)

$$P_{2}=1-P_{1}$$ (14b)

If both routes simultaneously reach their maximum allowable flow rates, then

$$v_{i}=\rho s_{i},\;i=1,2$$ (15)

Substituting Eqs (15) and (12) into Eq (14), we have

$$P_{1}=\Phi \left(\frac{\left(t_{2}^{0}-t_{1}^{0})(1+\tau \rho^{\beta }\right)}{\sqrt{\alpha (t_{2}^{0}+t_{1}^{0})(1+\tau \rho^{\beta })}}\right)=\Phi \left(\frac{(t_{2}^{0}-t_{1}^{0})\sqrt{\left(1+\tau \rho^{\beta }\right)}}{\sqrt{\alpha (t_{2}^{0}+t_{1}^{0})}}\right)$$ (16a)

$$P_{2}=1-\Phi \left(\frac{(t_{1}^{0}-t_{2}^{0})\sqrt{\left(1+\tau \rho^{\beta }\right)}}{\sqrt{\alpha (t_{2}^{0}+t_{1}^{0})}}\right)$$ (16b)

Based on Eq (16), we have

$$\frac{v_{1}}{v_{2}}=\frac{q\cdot P_{1}}{q\cdot P_{2}}=\frac{P_{1}}{P_{2}}\;$$

$$\Rightarrow \;\frac{s_{1}}{s_{2}}=\frac{\Phi \left(\frac{(t_{2}^{0}-t_{1}^{0})\sqrt{\left(1+\tau \rho^{\beta }\right)}}{\sqrt{\alpha (t_{2}^{0}+t_{1}^{0})}}\right)}{\Phi \left(\frac{(t_{1}^{0}-t_{2}^{0})\sqrt{\left(1+\tau \rho^{\beta }\right)}}{\sqrt{\alpha (t_{2}^{0}+t_{1}^{0})}}\right)}\;=\frac{\Phi \left(\frac{(t_{2}^{0}-t_{1}^{0})\sqrt{\left(1+\tau \rho^{\beta }\right)}}{\sqrt{\alpha (t_{2}^{0}+t_{1}^{0})}}\right)}{1-\Phi \left(\frac{(t_{2}^{0}-t_{1}^{0})\sqrt{\left(1+\tau \rho^{\beta }\right)}}{\sqrt{\alpha (t_{2}^{0}+t_{1}^{0})}}\right)}$$ (17)

Like it is demonstrated by Ge *et al*. [1], there are five cases to discuss according to Eq (17).

Case 1: $t_{1}^{0}>t_{2}^{0},s_{1}>s_{2}$
  In this case the slower route has greater capacity. This is a case that can be observed in every city, where the expressway are often regarded as a fast route with smaller capacity compared with a collection of parallel streets whose total capacity is larger but the average travel speed is lower. The network capacity in this case, however, cannot reach the maximum since it requires $\sqrt{\left(1+\tau \rho^{\beta }\right)}/\sqrt{\alpha (t_{2}^{0}+t_{1}^{0})}<0$. In fact, the better the quality (i.e., the smaller *α*) of the travelers’ information, the greater the underutilization of the capacity of the slower route. Therefore, the network capacity will decreases monotonously with respect to increased quality of travelers’ information.
Case 2: $t_{1}^{0}>t_{2}^{0},s_{1}<s_{2}$
  This case implies that the longer route also has smaller capacity. It is possible for the network capacity to reach their maximum allowable flow rates simultaneously, provided that
  $$\Phi \left(\frac{(t_{2}^{0}-t_{1}^{0})\sqrt{\left(1+\tau \rho^{\beta }\right)}}{\sqrt{\alpha (t_{2}^{0}+t_{1}^{0})}}\right)=\frac{s_{1}}{s_{1}+s_{2}}$$ (18)
  The corresponding α* which maximizes the network capacity can be calculated with Eq (18) by referring to the probability table of normal distribution. In this case, we can see that the larger the absolute difference between *α* and α* (i.e., |*α* − α*|), the greater underutilization of the capacity of the slower route. Therefore, the network capacity first increases monotonously with respect to increased quality of travelers’ information (decreased *α*) until it achieves the maximum value, and then it decreases monotonously in quality of travelers’ information.
Case 3: $t_{1}^{0}=t_{2}^{0},s_{1}=s_{2}$
  In this case, the Eq (17) is held definitely. Therefore, the network capacity is always the maximum value, and is independent with the level of travelers’ information.
Case 4: $t_{1}^{0}=t_{2}^{0},s_{1}\neq s_{2}$
  Under such conditions, Eq (17) does not hold, which implies there will always be unused capacity on the route with larger capacity.
Case 5:$t_{1}^{0}\neq t_{2}^{0},s_{1}=s_{2}$
  In this case, Eq (17) is hold only when *α* → + ∝. Thereby, the network capacity is a monotonic decreasing function with respect to level of travelers’ information.

For all the five cases listed above, the change pattern of the network capacity with respect to level of travelers’ information at Probit-based SUE condition is the same as it is demonstrated by Ge *et al*. [1] at Logit-based SUE condition. Besides, whether the network capacity can reach the maximum value is also the same at the two types of SUE condition. Consequently, we can draw a conclusion that for a general network, the variation pattern of network capacity with respect to level of travelers’ information at Probit-based SUE condition is the same as it is at Logit-based SUE condition, and if provided certain quality of travelers’ information, they can reach the same maximum value.

### 4.3 Numerical example 3

Numerical example 2 demonstrates with a small network that, under different distribution of link capacity and free flow travel time, the variation pattern of network capacity with respect to level of traveler information at Probit-based SUE is always the same as it is in Logit-based SUE. But in some very special cases, they may be different from each other. It is well known that there are two inherent drawbacks of the Logit model, i.e., (1) unable to account for overlapping (or correlation) among routes, and (2) unable to account for perception variance with respect to routes of different lengths. This two drawbacks may lead to significant different flow distribution outcome, and thus may result in different variation pattern of network capacity in traveler’ information level. The following loop-hole network will be used to demonstrate this claim.

**Fig 5:**
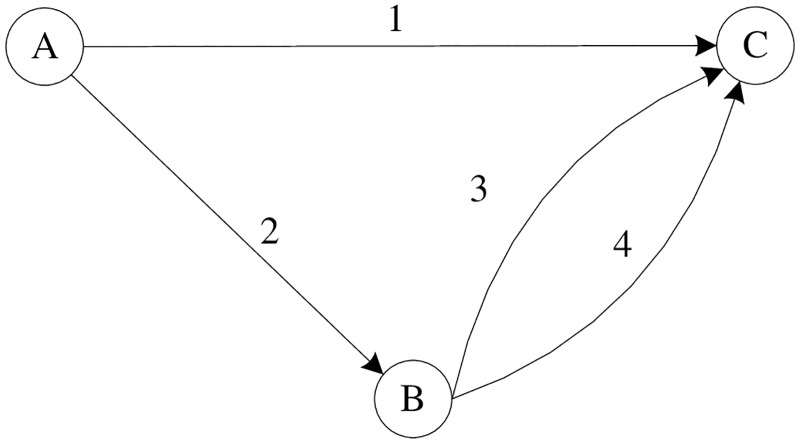
The loop-hole network.

The loop-hole network (Fig 5) is constantly used to illustrate the different traffic flow distribution between Probit-based SUE and Logit-based SUE. It only has one O-D pair (A-C) and three routes, i.e., route 1: link1; route 2: link 2-link 3 and route 3: link 2-link 4. The three routes all have the same free flow travel time and the capacity on link 2 is double of the capacity of other links. The network inputs are listed in Table 4. The free flow travel time on link 2, 3, 4 are assumed to be variable. The network capacity with Probit-based SUE problem can be formulated as Eq (5) without signal timing constraints (i.e., Eqs (2) and (4)). The initial O-D demand for O-D pair is 6, and the maximum saturation rate of all the links is set as 0.9.

**Table 4:** Input data for numerical example network.

Link *a*	1	2	3	4
Free flow travel time $t_{a}^{0}$	12	12 –*ζ*	*ζ*	*ζ*
Link capacity *s*_*a*_	8	16	8	8
Link travel time	$t_{a}(v_{a})=t_{a}^{0}{\left[1.0+0.15(v_{a}/s_{a}\right)}^{4}]$			

For this network, no matter what the free flow travel time on link 3 (i.e., *ζ*) and the Logit split *θ* are, the network capacity at Logit-based SUE state always stay at the maximum value it can obtain (i.e., 21.6) and route flow on the three routes are the same which is 7.2. The variation pattern of the network capacity with respect to travelers’ information in this network subjects to case 3 described in numerical example 2, that is, it is independent with the level of travelers’ information. It should note that the equilibrium route flow solution given by Logit-based SUE problem in this numerical example is not consistent with the real world. As *ζ* approaches to 0, the route 2 and route 3 merges into one route. Hence, the aggregate possibility to choose the two routes should be around 1/2. The Logit model, however, assigns one-third of the flow to each route regardless of the network topology, which overestimates the flow on the two overlapping routes. The unsatisfactory result occurs mainly because the Logit-based SUE can not address routes overlapping problem.

**Fig 6:**
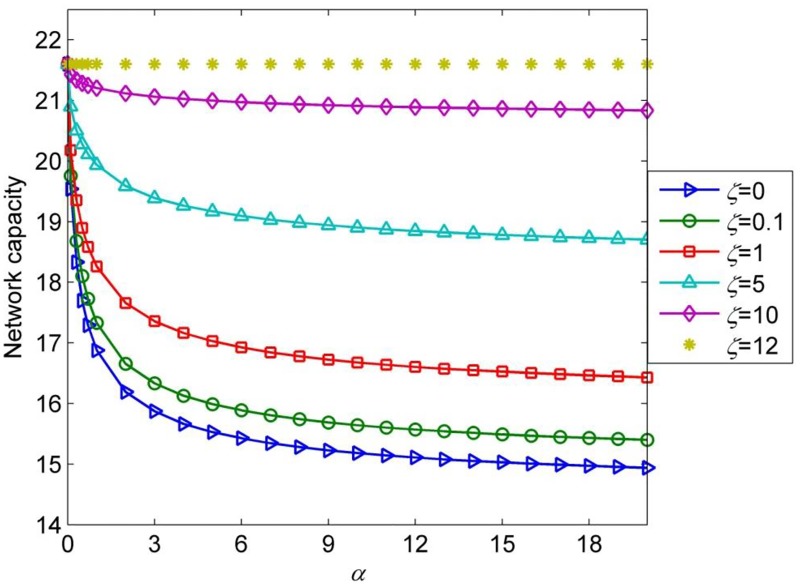
Network capacity with different *α* and *ς*.

**Fig 7:**
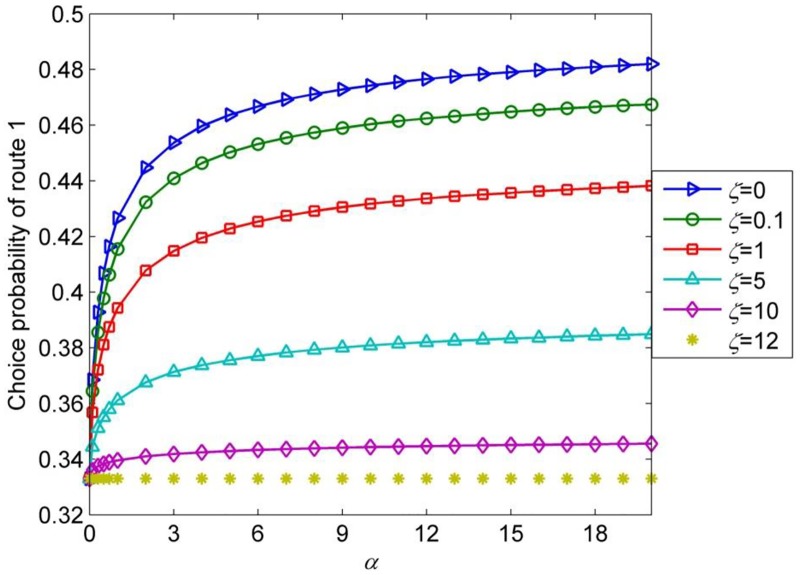
Probability of choosing route 1 with different *α* and *ς*.

In the following, we will discuss the network capacity at Probit-based SUE condition for this numerical network. The expected perceived link travel time is still assumed to be proportional to average link travel time with scale factor *α*. Fig 6 presents the network capacity with Probit-based SUE problem at different combinations of *α* and *ζ*. It clearly demonstrates that when the network degraded into three identical links (the probabilities to choose the three routes are the same in this scenario), the network capacity always remain the maximum value (i.e., 21.6) no matter how *α* changes. However, for fixed α, the network capacity decreases dramatically as *ζ* decreases. This is because the lower the *ζ*, the higher the probability that drivers would take route 2 and route 3 as one route. As a result, the probability to choose route 1 will increase (see Fig 7), and the flow that distributed to route 2 and route 3 reduces, thus the equilibrium link flows at Probit-based SUE condition cannot make full use of the physical link capacity. Fig 6 also denotes that for fixed *ζ*, *ζ* ≠ 0, the network capacity increases monotonously with respect to *α*, indicating that the network capacity at Probit-based SUE condition decreases as travelers’ information level increases. Consequently, the variation pattern of network capacity with respect to level of traveler’s information at Probit-based SUE condition in this numerical network is different from it is at Logit-based SUE condition.

The different variation pattern occurs because the Logit-based model lack of sensitivity to network topology, it assigns too much flow to the overlapped routes in the network. Recall that BPR function is an increasing function of the link flows, the route overlapping problem of Logit model can be greatly relieved in a congested network [28]. Therefore, for a general network, there should be no significant difference between the equilibrium route flows at Logit-based SUE and Probit-based SUE when maximum network capacity is reached. As a matter of fact, the numerical example network 3 is a very special network with idealistic input data. Test shows that once the link capacity can’t satisfy the relationship, i.e, *s*_1_ = *s*_3_ = *s*_4_ = 0.5*s*_2_, the variation pattern of network capacity with respect to level of travelers’ information at Probit-based SUE condition would be the same as it is at Logit-based SUE condition, and if provided certain quality of travelers’ information, they can achieve the same maximum value. Therefore, for general networks, although there may exist many overlapped routes, the variation pattern of network capacity with respect to travelers’ information at the two types SUE conditions will always be the same.

Experimental data presented in the above-mentioned figures and tables are obtained directly by running the program source code of the numerical experiment. In this study, the experiment program is written by Matlab 2010. The Matlab program source codes for numerical example 1 and 3 are saved on S1 and S2 Files, respectively. Experimental data for Fig 2, Tables 2 and 3 is saved on S3 File.

## 5. Conclusion

This paper studies the network capacity with Probit-based SUE problem. It is descripted as a bi-level programming problem, where the upper-level program is to maximize the network capacity through optimizing O-D multiplies and signal splits while the low-level program is the Probit-based SUE problem formulated to model the drivers’ route choice. A heuristic method based on sensitivity analysis for Probit-based SUE problem is explicitly presented to solve the bi-level network problem. Numerical applications on three example networks find that while network capacity could be different between Probit-based SUE and Logit-based SUE problem with some special network structure and inputs, the variation pattern of network capacity with respect to increased level of travelers’ information for general networks under the two SUE problems is the same. Besides, the maximum network capacity with both the Probit-based SUE and Logit-based SUE constraints can be the same under proper settings of level of travelers’ information. This study also finds that the network capacity cannot reach the maximum value when drivers have perfect knowledge of traffic condition, because better information allows a large portion of demand to use the fast route, thus saturates the weakest link of that route, making it impossible to accommodate more traffic.

This study can be used to find the optimal settings of network inputs to maximize network capacity, thereby mitigating the network congestion to certain extend. The analysis of three numerical example shows that, the impact of traveler’s information level on network capacity has no major difference using Probit-based SUE and Logit-based SUE problem. However, as it is hard to quantitatively decide the value of *α* and *θ* in Probit-based and Logit-based SUE problem, respectively, for given level of traveler’s information, we cannot explore how the different route choice behavior of the two SUE problems would impact network capacity. This problem will be examined in our future study.

## Supporting information

S1 File Program source code for numerical example 1. (TXT)

S2 File Program source code for numerical example 3. (TXT)

S3 File Experimental data for Fig 2 and Tables 2 and 3. (XLSX)
